# Attention modulates the effects of stimulus brightness and contrast on time perception

**DOI:** 10.3758/s13423-026-02893-9

**Published:** 2026-03-19

**Authors:** Hakan Karsilar, Hedderik van Rijn, Sebastiaan Mathôt

**Affiliations:** Department of Psychology, University of Groningen, Grote Kruisstraat 2/1, 9712TS Groningen, The Netherlands

**Keywords:** Attention, Precuing, Visual perception, Visual selective attention

## Abstract

The perception of duration is influenced by various factors, including attention and the physical properties of stimuli. However, the interaction between these factors remains poorly understood. The present study investigated how covert visual attention, stimulus brightness, and contrast jointly influence time perception using a novel attentional temporal bisection task. Participants categorized the duration of a cued target stimulus as “short” or “long” while the brightness and contrast (relative to background) of the target and a distractor stimulus were systematically manipulated. Results showed that high-contrast targets were perceived as lasting longer than low-contrast targets. Surprisingly, brighter targets were perceived as shorter than dimmer targets. At the unattended location, the effects of distractor contrast and brightness were the mirror image, such that targets were perceived as longer in the presence of bright distractors and (though not reliably) low-contrast distractors. Taken together, the findings point to local encoding effects at the target (contrast lengthens; higher target brightness shortens) and a modest decisional/context bias from the distractor, consistent with partial competition across locations. Findings further indicate that contrast, rather than absolute brightness, is the primary determinant of perceived duration, with its effects mediated by attention. This study provides novel insights into how bottom-up stimulus properties and top-down attention jointly influence temporal processing, highlighting the need for more nuanced models of time perception that account for the complex interplay between attention, brightness, and contrast. Finally, the counterintuitive finding that increasing brightness can shorten perceived duration challenges the notion of a monotonic relationship between stimulus intensity and perceived time.

**Supplementary Information:**

The online version contains supplementary material available at 10.3758/s13423-026-02893-9.

## Introduction

Time perception is a fundamental aspect of human cognition (Allman et al., 2014; Wittmann, 2013). Although humans are generally well able to estimate how long something lasts, perceived duration is systematically biased by non-temporal factors (Buhusi & Meck, 2005; Ivry & Schlerf, 2008; Lewis & Miall, 2003; Matthews & Meck, 2016). Specifically, two key factors that influence subjective time perception are attention and the physical properties of stimuli. However, how these factors interact remains unclear. The present study aimed to address this gap by systematically investigating the question: How do covert visual attention, stimulus brightness, and contrast interact to shape perceived duration?

Numerous studies have demonstrated that attended stimuli are perceived as lasting longer than unattended ones. This effect has been supported by studies employing attentional manipulations during timing tasks, such as cueing paradigms and dual-task designs. For instance, Enns et al. (1999) found that the perceived duration of a stimulus was longer when attention was directed to its location using a visual cue; similarly, Mattes and Ulrich (1998) showed that valid attentional cues prolonged the perceived duration of brief stimuli compared to invalid cues. Dual-task designs, where participants perform a primary timing task alongside a secondary task, also influence the perception of time. Brown (1997) demonstrated that dual-task conditions can distort the perceived duration, suggesting that competing cognitive demands impact temporal processing. Similarly, Fortin and Rousseau (1998) found that increased demands from a secondary task reduced the accuracy of timing judgments, indicating that resource allocation affects time perception (see also Hayashi et al., 2019; Ogden et al., 2021; Tse et al., 2004). Within the pacemaker–accumulator framework, often formalized with an attentional gate, directing attention to the to-be-timed stimulus allows a greater proportion (or effective rate) of pulses to reach the accumulator, lengthening perceived duration (Lejeune & Wearden, 2009; Zakay & Block, 1997). Overall, paying attention to a stimulus appears to systematically lengthen its duration. Notably, not all “attention” manipulations affect duration in the same direction. When attention is captured by highly arousing, semantically salient content (e.g., Tipples, 2010), perceived duration can be underestimated, consistent with resources being drawn away from the temporal-accumulation task, and towards other properties of the stimulus. We therefore distinguish between directing attention to the to-be-timed target (which should lengthen duration under an attentional-gate account) and attention being captured away from temporal processing by non-temporal content.

In addition to attention, the physical properties of the timed stimuli themselves can also significantly influence perceived durations, even in the absence of explicit attentional manipulations. Studies have consistently shown that stimuli with larger magnitudes in dimensions like size, numerosity, or speed tend to be perceived as having longer durations (Brown, 1995; Cai & Connell, 2015; Droit-Volet & Wearden, 2002; Eagleman, 2008; Herbst et al., 2013; Kanai & Watanabe, 2006; Kaneko & Murakami, 2009; Karşılar et al., 2018; Karşılar & Balcı, 2016, 2019; Matthews, 2011; New & Scholl, 2009; Ono & Kawahara, 2007; Otsuka & Yotsumoto, 2023; Rammsayer & Verner, 2014; Schlichting et al., 2020; Xuan et al., 2007; Yamamoto & Miura, 2012). Stimulus brightness, in particular, has been shown to lengthen perceived duration, with brighter stimuli judged as lasting longer than dimmer ones (Brigner, 1986; Goldstone et al., 1978; Goldstone & Goldfarb, 1964; Matthews et al., 2011). This effect is thought to stem from increased activation of the visual system in response to brighter stimuli (Matthews, 2011).

Recently, we found that the effect of brightness on time perception generalizes beyond just the timed stimulus itself. Specifically, the brightness of the background on which a stimulus is presented also dilates perceived duration (Karsilar et al., 2024). This finding raises two possibilities: the lengthening of perceived time could be due to (1) the overall higher luminance of the environment, or (2) the enhanced contrast between the timed stimulus and the background, as high-contrast stimuli are known to be more salient (Einhäuser & König, 2003; Itti & Koch, 2000; Turatto & Galfano, 2000). Some preliminary evidence supports an interpretation in terms of contrast. In tasks where the timed stimulus itself varies in contrast, higher contrast is associated with longer perceived durations (Eagleman, 2008; Matthews et al., 2011). Relatedly, Bruno and Johnston (2010) showed that contrast gain can shape visual time even when the timed interval’s contrast is held constant: by varying the *preceding* interval’s contrast (10% vs. 90%) while keeping the test interval at 50%, they observed robust changes in perceived duration, consistent with an early visual gain/adaptation mechanism rather than saliency differences at the timed interval. However, the extent to which the effects of stimulus brightness and contrast are mediated by covert visual attention remains an open question. A comprehensive investigation of how these factors interact is needed to clarify the mechanisms underlying temporal distortions.

The current study aimed to address this gap in the literature by systematically investigating the effects of stimulus brightness and contrast on time perception while considering their interaction with attention. To that end, we employed a novel task that combines temporal bisection with attentional cueing. In this modified temporal bisection task, participants categorize the duration of a cued target stimulus as “short” or “long” based on previously learned reference durations. Our paradigm builds upon previous work using cues to manipulate attention in timing tasks (Enns et al., 1999; Mattes & Ulrich, 1998), but crucially extends this approach to systematically investigate the interaction between attention, stimulus brightness, and contrast. By systematically manipulating the contrast of target (attended and to-be-timed) and distractor (unattended and to-be-ignored) stimuli relative to the background, we aimed to provide a comprehensive understanding of how these factors interact to influence time perception. Crucially, we held the background luminance constant and selected four target and distractor luminance levels that were symmetrically spaced in physical luminance (and log units) around the background.

Given previous work, we hypothesized that (1) brighter targets will be perceived as lasting longer than dimmer targets, and (2) high-contrast targets will be perceived as lasting longer than low-contrast targets. Regarding the effects of the brightness and contrast of the distractor, we considered two competing hypotheses. One possibility is that a brighter or higher-contrast distractor could cause the target to appear as lasting longer, which would suggest that the effects of brightness and contrast on perceived duration are general low-level phenomena that are not mediated by covert visual attention. Alternatively, a brighter or higher-contrast distractor might cause the target to appear as lasting for a shorter time by drawing attention away from the target stimulus. This would indicate that the effects of brightness and contrast are mediated by attention, with a reduction in attention to the target leading to a shorter perceived duration. These two hypotheses lead to divergent predictions, and we did not favor one over the other a priori, but rather sought to adjudicate between them empirically. The findings of this study will contribute to a more nuanced understanding of how bottom-up stimulus properties and top-down attention jointly shape temporal processing, with potential implications for refining existing models of time perception.

## Methods

### Participants

Sixty-five participants (43 female, *M*_age_ = 20.46 years) participated in the experiment. Participants were recruited via the University of Groningen psychology participant pool and enrolled through the department’s online sign-up system in exchange for course credit. The study was exempt from full ethical review (approval code: PSY-2324-S-0020) and was conducted in compliance with the ethical guidelines set by the Faculty of Behavioural and Social Sciences for human subjects’ research. All participants provided written informed consent prior to the study and received course credit for their contribution. All participants had normal or corrected-to-normal vision. Two participants were excluded from the study due to unusual response times, which suggested insufficient engagement with the task (see *Results* section below). In the absence of an expected effect size, sample size was based on previous similar studies on temporal perception (Penney & Cheng, 2018; Wearden & Ferrara, 1995).

### Stimuli and apparatus

**Fig. 1:**
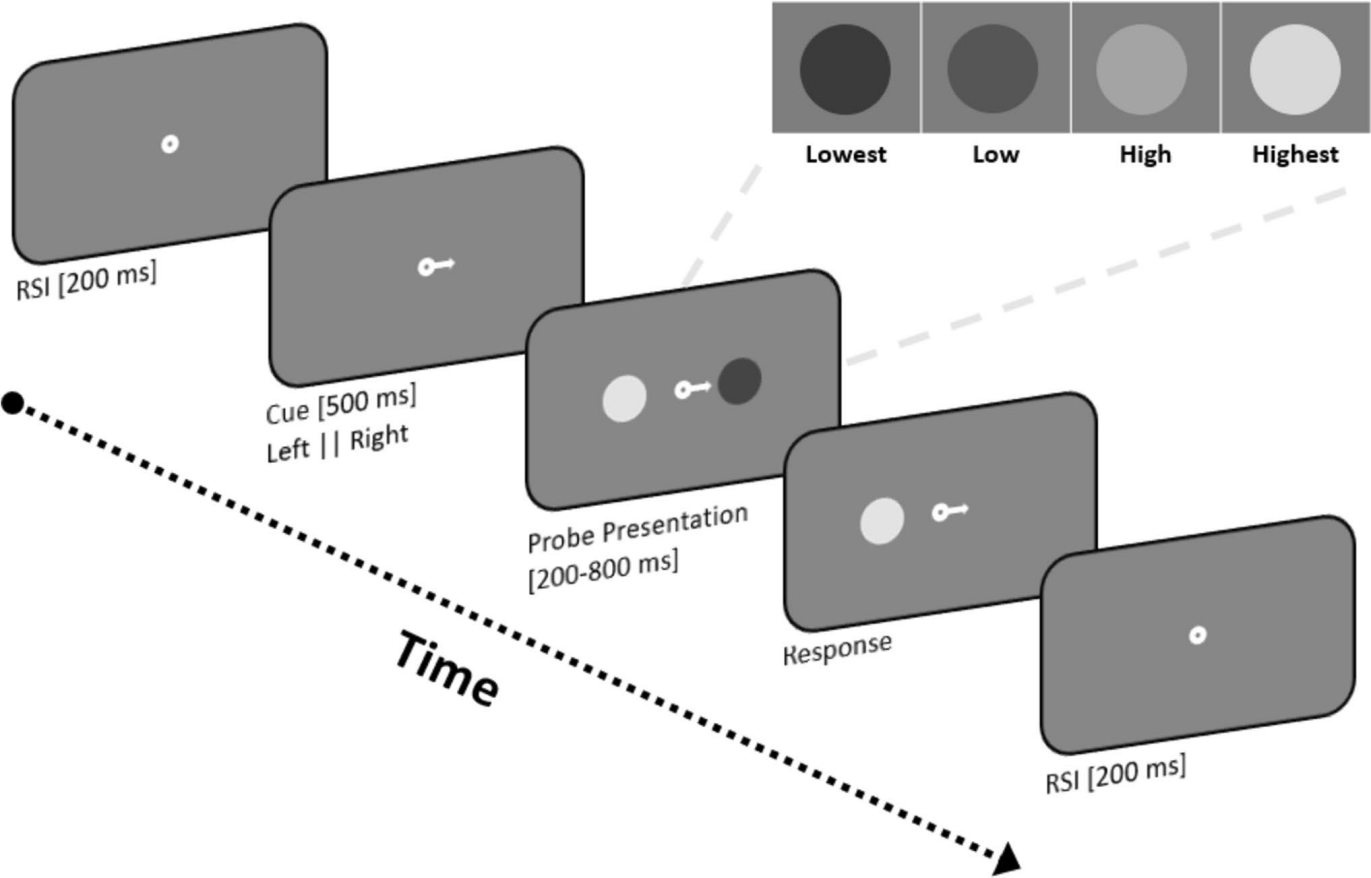
Timeline of a single trial in Experiment 1. Inset depicts four levels of stimulus brightness. See text for details

Stimuli were presented on a 27-in. LCD monitor (1,920 × 1,080; 60 Hz; Brand: Iiyama ProLite - G2773HS) using OpenSesame 3.3 (Mathôt et al., 2012; Mathôt & March, 2022) with PsychoPy (Peirce, 2007) for stimulus presentation and PyGaze (Dalmaijer et al., 2014) for eye tracking. Visual updates were synchronized to the display’s vertical refresh, and stimulus durations were specified as integer multiples of the 16.67-ms frame period. Stimulus onsets and offsets were abrupt (square-wave), occurring on a single refresh without any gradual contrast ramp; luminance remained constant for the entire on-screen duration. An EyeLink 1000 (SR Research) recorded the right eye at 1,000 Hz. Participants sat ~60 cm from the monitor with head stabilized in a chinrest, in a dimly lit room, and responded via a wired keyboard. The stimuli were filled circular patches (target and distractor), each subtending 2.39° of visual angle. Each trial began with an attentional cue (arrow; 0.86°). All visuals appeared on a constant background of 60.53 cd/m^2^ (see Fig. 1). To manipulate target and distractor brightness, we used four luminance levels (see *Luminance and contrast*).

#### Luminance and contrast

Luminance was measured with an on-screen colorimeter (Datacolor Spyder4Pro) placed against the display; measurements were taken in a dimly lit room before data collection, and the Spyder software reported cd/m^2^. The measured gray background was 60.53 cd/m^2^. We selected four target luminances (33.38, 44.70, 81.79, and 109.50 cd/m^2^) symmetric around the background in physical and logarithmic units (Weber–Fechner spacing). This way, the Michelson contrast [C_M_ = (L_target_ − L_background_)/(L_target_ + L_background_)] of the highest (109.50 cd/m^2^) and lowest (33.38 cd/m^2^) luminances was identical (C_M_ = ±0.29), as was the Michelson contrast of the intermediate high (81.79 cd/m^2^) and intermediate low (44.70 cd/m^2^) luminance levels (C_M_ = ±0.15).

The contrast of a single uniform patch on a uniform background can be described in different ways. Here we consider three ways to describe the contrast of our patches. In the *Statistical analysis *section, we describe which of these contrast measures was selected for analysis, and why. In *binary contrast coding*, contrast was simply coded as low or high. In other words, the binary contrast coding reflects that there were only two different levels of Michelson contrast. In *continuous contrast coding*, contrast was coded as the absolute luminance difference from background, |ΔL| = |L_target_ − L_background_|. Finally, in *Weber contrast coding*, contrast was defined using Weber contrast [C_W_ = (L_target_ − L_background_)/L_background_)], resulting in four values: −0.45, −0.26, 0.35, and 0.81. Since our luminance values were chosen with Michelson contrast in mind, we only provide Weber contrast for reference without considering it as a possible coding for analysis.

### Procedure

Each experimental session consisted of a training block followed by a test block, and lasted approximately 50 min. Each session started with a standard 5-point tracking calibration with white targets. Each trial within a session was initiated with a drift correction (single-point recalibration) procedure. Prior to training, participants were informed that they would be participating in a time-perception experiment where they would categorize durations as “short” or “long”; duration was defined as the amount of time that a circle stays on the screen. Participants were required to press the “F” and “J” keys with their index fingers to report a “short” or a “long” duration. The mapping of the keys was counterbalanced across participants. Participants were instructed not to count or use any other chronometric methods such as tapping throughout the experiment. Participants were told that the purpose of the eye tracker was to ensure that they always focused on the central fixation dot throughout the experiment.

### Attentional temporal bisection task

The temporal bisection task entails categorizing durations as “short” or “long” based on their similarity to previously learned reference durations. As such, each session began with a brief training block where two reference durations were presented with equal likelihood, and learning was demonstrated by 20 correct categorizations of the two reference durations (200.4 and 801.6 ms). Visual feedback was given for correct and incorrect responses (700 ms). The majority of the participants required fewer than four correction trials. The test block was nearly identical to the training block, except for the exclusion of accuracy feedback, and the presentation of stimuli for a duration randomly selected among six probe durations (200.4, 317.3, 434.2, 567.8, 684.7, and 801.6 ms).

Each trial started with a 200-ms fixation dot followed by an attentional cue, randomly pointing to the right or the left side of the screen. The cue signaled the side on which the target (i.e., the to-be-timed stimulus) would appear. 500 ms following the cue onset, two filled circles representing target and distractor (2.39° diameter) were simultaneously presented on both sides of the screen centered at a distance of 2.76° from the fixation dot. The brightness of the stimuli was manipulated trial-to-trial (four levels; Lowest, Low, High, Highest). The target stimulus disappeared after one of the two reference durations in the training block, and one of the six probe durations in the test block. The attentional cue and distractor stimulus stayed on the screen until a response was given. Each response was followed by a blank screen of 200 ms. Target and distractor luminance levels were selected independently and fully crossed on each trial (4 × 4 combinations), with no constraints linking the two streams. The test block consisted of 384 trials (Probe Duration (6 levels) × Cue Direction (2 levels) × Target Brightness (4 levels) × Distractor Brightness (4 levels) × 2 repetitions).

### Analytical approach

In traditional psychophysical approaches to temporal bisection, Point of Subjective Equality (PSE) is often taken from the 50% point of a fitted psychometric function (e.g., sigmoid or Weibull curves) based on response proportions (Allan & Gibbon, 1991; Karşılar et al., 2018; Karşılar & Balcı, 2019). Lower (leftward) PSEs indicate faster subjective time (Meck, 1996). The downside of this approach is that it requires a large number of observations for each point used to fit the psychometric function; in our case, this would mean a large number of trials for each unique combination of participant, duration, target brightness, and distractor brightness. This would require a very long experiment.

Therefore, we instead used generalized linear mixed-effects models (GLMMs) that predict each response (long/short) as a logistic function of probe duration and other fixed effects (experimental factors). GLMMs retain all trial-level data, accommodate occasional imbalance or missing trials, naturally handle binomial outcomes (long/short), and capture between-participant variability via random effects. GLMMs are a more statistically powerful alternative to traditional psychometric functions.

Although our main analyses focus on GLMMs, this approach also allows us to determine PSEs. We therefore complemented our main analyses with a set of PSE analyses that are closer to the traditional approach of fitting psychometric functions. PSEs can be derived as follows: For any fixed combination of luminance/contrast factors, GLMM defines a logistic psychometric function over duration:

$${\mathrm{log}}_{\mathrm{it}}\left\{P\left(\left.\mathrm{Long}\right|\mathrm{duration},\mathbf{x}\right)\right\}=\beta 0+\beta 1\text{ duration}+{\beta }^{\mathrm{T}}\mathbf{x}+{u}_{0,\mathrm{subj}}+{u}_{1,\mathrm{subj}}\boldsymbol{ }\mathrm{duration}$$

Here, duration corresponds to the duration, and x corresponds to the other fixed effects (predictors) in the model. *u*_1,subj_ corresponds to the participant-specific random intercept, or, phrased differently, a participant’s overall response bias. *u*_0,subj_ is an optional parameter that, when present, corresponds to the participant-specific random slopes for the fixed effects; for example, if a participant is more strongly affected by target contrast than the average participant, then there would be a positive random slope for target contrast for that participant. Importantly, for a given combination of fixed effects (x), we can derive the duration at which *P*(Long) = 0.5 from the model. This duration corresponds to the PSE.

## Results

Eye-tracking data were preprocessed using the Python eyelinkparser package (Mathôt & Vilotijević, 2022). Each participant completed 384 test trials. Trials with reaction times shorter than 100 ms and longer than the subject’s mean RT + 3*SD were removed. Trials on which horizontal eye position deviated from the central fixation dot by more than 1.62° (i.e., the distance to the inner edge of the target and distractor) were removed from further analyses (3.61% of all trials). Trials were usable if a binary response was recorded and all quality criteria were met (valid RT bounds, non-practice, no timeouts/technical errors). Participants with < 80% usable trials were removed; two of 65 were excluded by this rule (final *N* = 63).

### Statistical analysis

The analyses described in subsequent sections all rely on a single statistical model. This section describes why we chose this model over alternatives in a data-driven way.

A first analytical choice is between random-intercept-only models, which assume that there are no individual differences between participants except for a constant response bias (the intercept), and random-intercept-and-slope models, which assume that there are individual differences between participants. Random slopes are generally preferred, but are not always feasible because the resulting models are often too complex to be properly fitted. A second analytical choice is between two different possible ways of coding contrast: *binary contrast coding* or *continuous contrast coding* (see *Luminance and contrast*). In total, we thus considered four (2 × 2) candidate model configurations.

For each candidate model configuration, we determined the AIC (Akaike Information Criterion), as shown in Table 1, where lower AIC values are better. The model configuration with the lowest AIC value used binary contrast coding and included random slopes. Therefore, the resulting base model was a binomial-logit GLMM with response (long/short) as dependent measure; binary contrast (high or low), binary brightness (brighter or darker than background), and probe duration (mean-centered to reduce collinearity between the intercept and the slope, improving numerical stability) as fixed effects, and by-participant random intercepts and slopes for all fixed effects. Crucially, this coding follows the design logic of placing luminance levels symmetrically around the constant background to disentangle directional brightness from contrast magnitude, and it avoids imposing an ordinal slope on contrast, which would be inappropriate here because the absolute-contrast ordering is non-monotonic across the four luminance levels. The base model did not include any interactions.

**Table 1:** AIC (Akaike Information Criterion) comparison of four generalized linear mixed-effects models (GLMMs) crossing contrast coding (binary vs. continuous ∣ΔL∣) with random-effects structure (intercept-only vs. random slope for duration)

Contrast coding	Random effects structure	AIC	ΔAIC vs. best
Binary	Random slope (*1 + duration | subject*)	16909.00	0.00
Continuous |ΔL|	Random slope (*1 + duration | subject*)	16914.92	5.92
Binary	Intercept-only (*1 | subject*)	17215.11	306.11
Continuous |ΔL|	Intercept-only (*1 | subject*)	17221.00	312.00

All models were fit using the glmer function in the lme4 package (Bates et al., 2015) in R (R Core Team, 2020).

### Robustness of the generalized linear mixed-effects model (GLMM)

We note that the logistic psychometric function assumed by our GLMM is not an arbitrary choice but the theoretically predicted response function for temporal bisection under scalar timing theory (Allan & Gibbon, 1991; Gibbon, 1977). Importantly, although the fully crossed design yields only four trials per design cell, the random effects in our model (by-participant intercept and duration slope) are each informed by all ~384 trials from a given participant, not by individual cells. To verify the robustness of this approach, we conducted three supplementary analyses. First, a parameter recovery simulation (100 Monte Carlo iterations using our exact design) demonstrated that the GLMM reliably recovers participant-level random intercepts (median r =.95) and duration slopes (median r =.93; Online Supplementary Material (OSM) Section S1). Second, participant-level psychometric-function plots confirmed that all 63 participants show well-behaved sigmoid response patterns consistent with the logistic link function (OSM Section S2). Third, a leave-one-subject-out analysis on the full manuscript model showed that all six fixed-effect coefficients were highly stable across all 63 iterations (e.g., the duration coefficient showed a maximum deviation of 1.2%; OSM Section S3). Lastly, the estimated standard deviation of the random intercept was τ_intercept_ = 1.01 (on the log-odds scale), and the standard deviation of the random duration slope was τ_slope_ = 3.20, indicating substantial between-participant variability in both overall response bias and temporal sensitivity (psychometric steepness), indicating substantial individual variability that is well handled by the GLMM’s partial pooling mechanism.

### Higher target contrast and distractor brightness lengthen perceived time

#### Main results

The probability of a “long” response increased steeply with duration (centered seconds; β = 12.259, *SE* = 0.434, *p* <.001), reflecting that participants indeed perceived longer durations as longer. Higher target contrast increased the probability of a “long” response (β = 0.099, *SE* = 0.020, *p* <.001), reflecting high-contrast targets were perceived as longer. Higher target brightness decreased long responses (β = −0.085, *SE* = 0.020, *p* <.001), reflecting that bright targets were perceived as shorter. Higher distractor contrast numerically reduced long responses, although this effect was not significant (β = −0.006, *SE* = 0.020, *p* =.775). Higher distractor brightness increased long responses (β = 0.048, *SE* = 0.020, *p* =.013), reflecting that targets were perceived as longer in the presence of a bright distractor.

#### Interactions

To assess potential interactions between brightness sign and contrast (absolute luminance difference from the background), we fit five GLMMs that each added a single interaction to the additive base model:

1. Target brightness sign × target contrast,
2. Distractor brightness sign × distractor contrast,
3. Target brightness sign × distractor brightness sign,
4. Target contrast × distractor contrast, and
5. An omnibus model containing all four interactions.

All models used the same random-effects structure (1 + duration | subject). None improved fit relative to the additive model by likelihood-ratio tests or information criteria (all *p*s >.50). We therefore retain the additive random-slope GLMM as the primary model.

#### PSEs

**Fig. 2:**
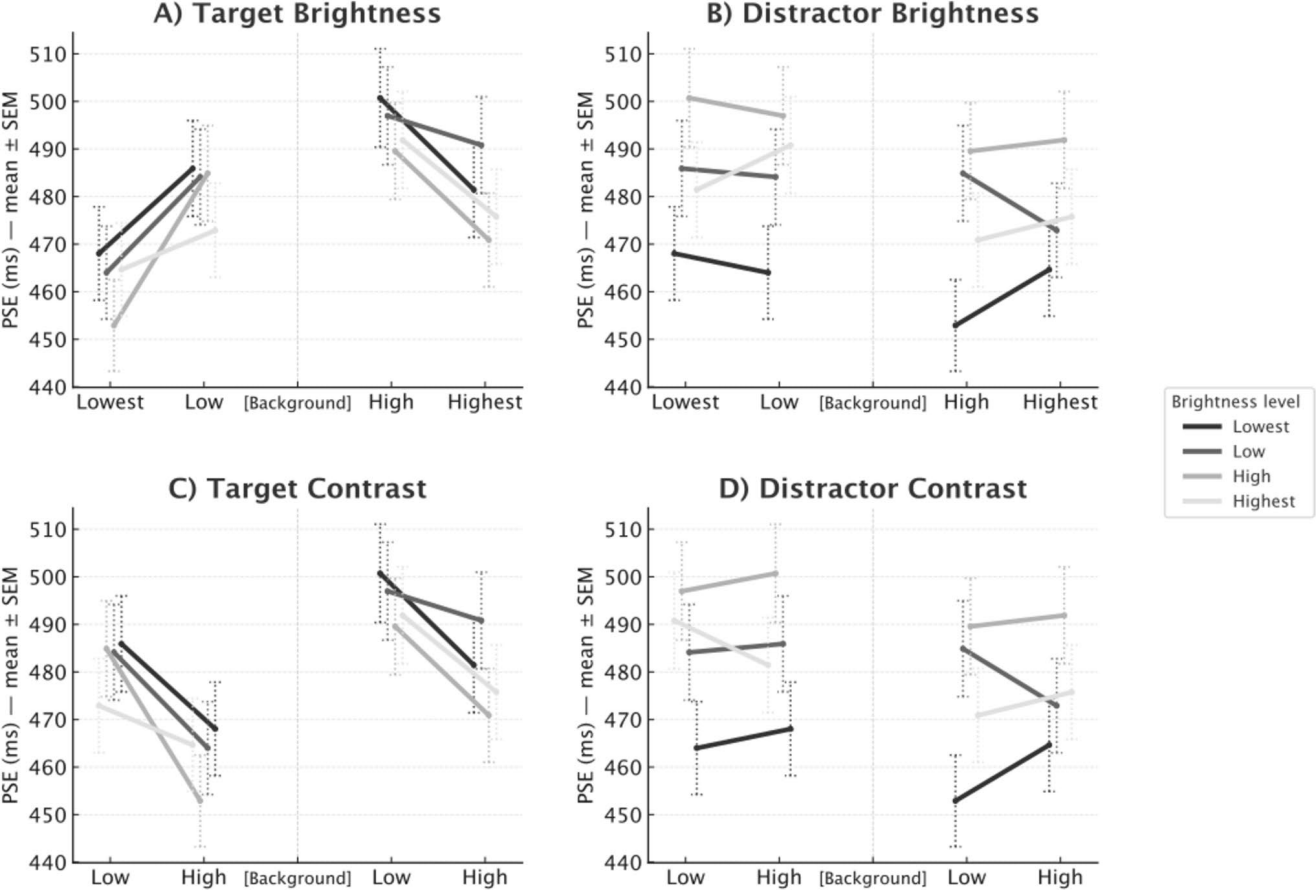
Group Points of Subjective Equality (PSEs) (ms) for cued and uncued brightness/contrast, arranged to visualize target–distractor differences. In each panel, curves hold one stimulus fixed (lines) while the other varies across the x-axis, so vertical gaps between curves at a given x-level reflect how *differences* between target and distractor map to PSE. Brightness (**A, B**): x-axis is 1–2–[Background]–4–5. Contrast (**C, D**): the same axis is used, but each side is shown Low→High within its pair (left: 2→1; right: 4→5) to allow side-by-side comparison of difference-based trends. Mapping: all line tones and icons are grayscale and match the actual stimulus levels (1 = darkest … 5 = lightest). Points/lines depict group means; dashed vertical whiskers show ±1 SEM (thin caps) computed from subject-level PSEs. PSEs were derived from the selected generalized linear mixed-effects model (GLMM) with a random intercept and a random duration slope by subject

As a secondary analysis, we also analyzed PSEs (see *Analytical approach*). The group-level PSEs from the selected random-slope model reveal a clear contrast-driven dilation at the target location (Fig. 2). At the target, increasing contrast from Low to High shifted the PSE leftward by 24 ms, consistent with longer perceived durations at higher contrast (Low: 485 ms, CI [466, 504]; High: 461 ms, CI [443, 479]). At the distractor, increasing contrast from Low to High produced a small rightward shift of +2 ms (Low: 470 ms, CI [452, 489]; High: 472 ms, CI [454, 490]).

**Fig. 3:**
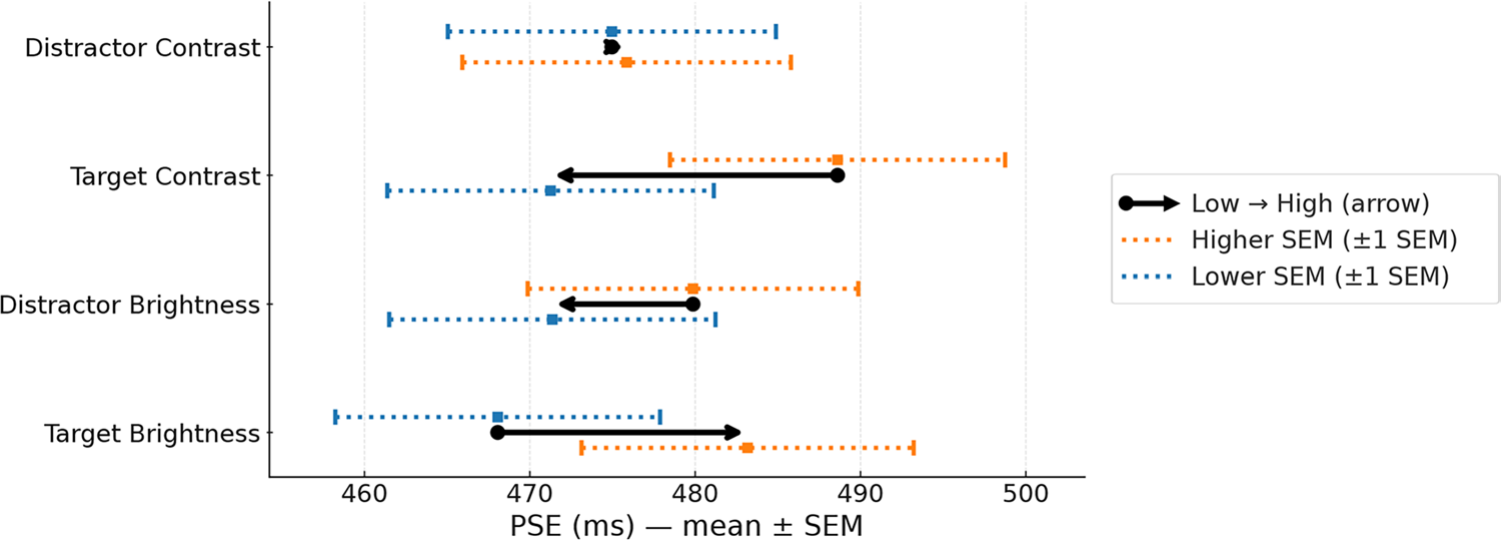
For each row (Distractor Contrast, Target Contrast, Distractor Brightness, Target Brightness), the circle marks the group mean Point of Subjective Equality (PSE) at the Low level and the arrow shows the shift from Low → High. Dotted horizontal whiskers depict ±1 SEM with short vertical caps; a small square marks each whisker’s center. Contrast rows: the named stimulus’s contrast is set to Low (0.0) or High (1.0); the brightness sign for both stimuli is neutral (coded 0, i.e., averaged over darker/brighter), and the other stimulus’s contrast is fixed at its sample mean. Brightness rows: the named stimulus’s brightness sign is Below vs. Above background; both stimuli’s contrast| are fixed at their sample means. PSEs were obtained per participant from the generalized linear mixed-effects model (GLMM) (*1 + duration | subject*) as the duration with *P*(Long) = 0.5 and then averaged across participants. (Whisker colors distinguish the larger vs. smaller SEM only; the stimuli were grayscale)

Overall, brightness showed opposite effects across locations: At the target, increasing brightness from Below to Above background shifted the PSE rightward by 25 ms, consistent with shorter perceived durations (Below: 459 ms, CI [441, 477]; Above: 484 ms, CI [465, 502]). Conversely, at the distractor, increasing brightness from Below to Above background shifted the PSE leftward by 9 ms, consistent with longer perceived durations (Below: 476 ms, CI [457, 494]; Above: 467 ms, CI [448, 485]). Figure 3 summarizes the shifts in PSEs visually.

### Sensitivity (Weber ratio)

To assess whether PSE shifts were accompanied by changes in temporal sensitivity, we derived JNDs (just noticeable differences) and Weber ratios (WR = JND/PSE) from the random-slope GLMM by locating *P*(Long) =.25, *P*(Long) =.50, and *P*(Long) =.75 for each combination of participant and condition. WRs were stable across conditions (≈ 0.20), indicating that PSE effects were not driven by sensitivity changes (Table 2).

**Table 2:** Mean Weber ratios (WR = JND/PSE) and 95% confidence intervals across experimental conditions

Factor	Level	WR mean	95% CI	Δ (high−low/above−below)
Distractor contrast	Low	0.205	[0.192, 0.217]	−0.0004
	High	0.204	[0.192, 0.216]	
Target contrast	Low	0.201	[0.189, 0.212]	+0.0080
	High	0.209	[0.196, 0.221]	
Distractor brightness	Below	0.203	[0.191, 0.215]	+0.0039
	Above	0.206	[0.194, 0.219]	
Target brightness	Below	0.208	[0.195, 0.221]	−0.0069
	Above	0.201	[0.189, 0.213]	

*JND* = just noticeable difference, *PSE* = Point of Subjective Equality

## Discussion

The present study investigated how covert visual attention, stimulus brightness, and contrast interact to shape perceived duration. Our cued attentional manipulation aimed to influence perceived durations as a function of the brightness and contrast of the target and the distractor. This manipulation was based on previous literature where increased attention is thought to lead to a more efficient accumulation rate in a hypothetical internal clock mechanism (Gibbon et al., 1984), resulting in longer perceived durations (Brown, 1997; Macar et al., 1994; Matthews & Meck, 2016; Tse et al., 2004; Zakay & Block, 1996). Consistent with this mechanism, the data point to two partly dissociable influences on perceived duration: a stimulus-driven effect at the attended target and a modest decisional bias from the unattended distractor. At the target, increasing brightness compressed perceived duration, whereas increasing contrast lengthened it. At the distractor, increasing brightness lengthened perceived duration – mirroring its effect at the target – and increasing contrast biased the responses toward “short,” although the effect was weak and statistically uncertain. Overall, our results suggest that contrast, rather than brightness per se, is the main determinant of perceived duration.

The lengthening effect of target contrast aligns with prior work in which increasing the timed stimulus’s contrast lengthened perceived duration (Eagleman, 2008; Matthews et al., 2011). Complementing this, Bruno and Johnston (2010) demonstrated that contrast gain can modulate perceived time even when the test interval’s contrast is fixed (50%): adjusting the *preceding* interval’s contrast (10% vs. 90%) altered perceived duration, consistent with early visual gain/adaptation rather than saliency at the timed interval. This effect may be attributed to enhanced visual salience and faster attentional processing of high-contrast stimuli (Einhäuser & König, 2003; Itti & Koch, 2000; Turatto & Galfano, 2000), hypothetically due to more efficient accumulation of time-representative pulses in pacemaker-accumulator models (Lejeune & Wearden, 2009; Zakay & Block, 1997). The unexpected finding that brighter targets were perceived as shorter than dimmer targets appears to contradict previous research (Brigner, 1986; Goldstone et al., 1978; Goldstone & Goldfarb, 1964; Matthews et al., 2011). However, the brightness pattern – which was negative at the attended target and positive at the unattended distractor – can also be interpreted as a contrast-based account rather than a pure brightness effect: if, in our display, above-background (“brighter”) targets were more similar to the background than below-background (“dimmer”) targets, the target would be less salient and capture less attention, shortening perceived duration. We did not psychophysically measure perceived distances to the background nor collect spontaneous similarity reports; we therefore present this as a plausible mechanism rather than as a measured fact (see *Methods: Luminance and contrast*).

Our results suggest that the effects of the brightness and contrast of the distractor were essentially the mirror image of these same effects for the target, given that the effect of distractor contrast was also in the opposite direction of the target, despite not reaching statistical significance. Similar to the effect of target brightness, the effect of distractor brightness on perceived target duration is likely driven primarily by contrast rather than absolute brightness. Taken together, our results suggest that stimulus properties that make the target more salient, such as high contrast, increase perceived target duration, while properties that capture attention away from the target, such as a salient distractor, reduce perceived target duration.

Our findings also extend prior evidence that covert attention alters perceived duration depending on the features to which attention is directed. For instance, Hayashi et al. (2019) demonstrated this with moving stimuli, where motion and direction can both engage attention (see also for *motion*: Kanai et al., 2006; Kaneko & Murakami, 2009; Tomassini et al., 2011; *attention*: Casini & Macar 1997; Coull et al., 2004; Tse et al., 2004). We manipulated contrast independently from brightness. Our results indicate that feature-based gain in early visual coding – here, via contrast – can modulate subjective time without motion. In this sense, contrast provides a theoretically informative complement to motion: it (i) avoids confounding temporal dynamics intrinsic to moving stimuli, (ii) connects directly to well-studied salience and contrast-gain mechanisms, and (iii) shows that the attentional influence on duration generalizes from motion features to luminance contrast, strengthening a feature-general (not motion-specific) account of attentional time dilation.

One open question raised by our findings is whether brightness and contrast at an unattended location could potentially exert dissociable effects on perceived duration. Specifically, in the case where a brighter but lower-contrast distractor lengthens the perceived duration of the target stimulus, the increased brightness of the distractor might enhance its salience, leading to a lengthening of perceived time, even though its lower contrast reduces its attentional capture. This interpretation suggests that brightness and contrast at an unattended location could have dissociable effects on time perception, with brightness influencing perceived duration through low-level mechanisms, while contrast affects time perception primarily through its impact on attention. However, a more plausible interpretation of our findings is that brightness levels might not have been perceptually equidistant from the background according to Weber-Fechner’s Law. Future studies can explore the idea of potentially dissociable effects of brightness and contrast by using more sophisticated methods to control for perceptual distance, such as adaptive staircase procedures or individual perceptual matching tasks.

### Limitations

There are some limitations to consider in our study. First, we used a limited range of stimulus durations (200–800 ms) and brightness levels; future research could explore a wider range of durations and luminance values to test the generalizability of our findings (see Buhusi & Meck, 2005; Lewis & Miall, 2009). Second, we used simple geometric stimuli (filled circles); it would be interesting to investigate whether our results extend to more complex and ecologically valid stimuli, such as natural scenes (Davydenko & Peetz, 2017) or faces (Fayolle & Droit-Volet, 2014). Lastly, our study did not include a neutral condition without attentional cueing. Such a condition would allow for a more direct comparison of the effects of brightness and contrast on attended versus unattended stimuli. Future research could include this condition to provide a more complete picture of how these factors interact. Additionally, the fixation dot was black, making it visually conspicuous against the gray background. Although the fixation dot was spatially separated from the stimuli (~2.76° from the inner edge of the patches), it may have served as a local brightness or contrast anchor, potentially influencing the perceived brightness or contrast of nearby stimuli. Future studies could use a fixation dot matched in luminance to the background, or adopt a ring-shaped fixation marker, to rule out any such influence.

## Conclusion

In conclusion, our study makes a valuable contribution by highlighting the complex interplay between attention, brightness, and contrast in shaping time perception, providing novel insights into how bottom-up stimulus properties and top-down attention jointly influence perceived duration. Crucially, our findings challenge the notion of a unified common magnitude system (Bueti & Walsh, 2009; Walsh, 2003) where “brighter stimuli lead to longer perceived time,” and underscore the need for more nuanced models that account for the differential effects of various stimulus properties on temporal processing. Our results extend this literature by demonstrating that such effects hold even when attention is manipulated through cueing and that the contrast of an unattended stimulus can influence the perceived duration of an attended stimulus. This suggests that the effects of contrast on time perception may operate at a lower level than the effects of endogenous attention. Additionally, our findings have potential practical implications for professionals in fields where accurate temporal judgments are critical, such as aviation, sports, and medicine. For example, our results suggest that the brightness and contrast of visual displays could be optimized to minimize temporal distortions and improve performance in time-sensitive tasks. Specifically, high-contrast warning signals or signage may be perceived as lasting longer, potentially increasing their effectiveness, which could have implications for tasks requiring precise timing. In sum, our study opens up new avenues for future research and has potential practical implications for professionals in fields where accurate temporal judgments are essential.

## Supplementary Information

Below is the link to the electronic supplementary material.

Supplementary file1 (DOCX 978 KB)

## Data Availability

The datasets generated during study, the experiment script and experiment materials are available in the Open Science Framework repository, at https://osf.io/k8uqw/.

## Code availability

The data analysis scripts are available in the Open Science Framework repository, at https://osf.io/k8uqw/.
