# Supplementary material for: Attention modulates the effects of stimulus brightness and contrast on time perception
Source: Psychon Bull Rev. 2026 Mar 19;33(4):118. doi: 10.3758/s13423-026-02893-9 (PMC13002725; doi:10.3758/s13423-026-02893-9)
Supplement: Supplementary file 1 — Supplementary file1 (DOCX 978 KB) [file 13423_2026_2893_MOESM1_ESM.docx]

**Supplementary Materials**

*Attention Modulates the Effects of Stimulus Brightness and Contrast on Time Perception*

Hakan Karsilar, Hedderik van Rijn, and Sebastiaan Mathôt

This document presents three supplementary analyses that address concerns about the reliability and robustness of the generalized linear mixed-effects model (GLMM) used in the manuscript. All analyses can be reproduced using the accompanying R script (Supplementary_Analysis.R), available at the OSF repository.

## S1. Parameter Recovery Simulation

There may exist the concern that, with only four trials per design cell, participant-level estimates may be unreliable. Crucially, in our GLMM, the random effects (by-participant intercept and duration slope) are each informed by *all* ~384 trials from a given participant, not by any single design cell. A single miscoded response thus affects only 1/384 = 0.26% of a participant’s data, and partial pooling further attenuates its influence. To formally demonstrate that the GLMM reliably recovers individual random effects under our exact design, we conducted a Monte Carlo parameter recovery simulation.

***Methods***

We simulated 100 synthetic datasets, each mimicking our experimental design: 63 participants × 384 trials (6 probe durations × 4 target brightnesses × 4 distractor brightnesses × 2 cue directions × 2 repetitions). For each dataset, participant-specific random intercepts and duration slopes were drawn from a bivariate normal distribution with these parameters; τ_intercept_ = 0.50, τ_slope_ = 3.00, ρ = −0.30. These simulation parameters are deliberately more conservative than the empirical estimates (τ_intercept_ = 1.01, τ_slope_ = 3.20, ρ = +0.40; see S3), meaning the simulation tests a harder scenario: with smaller between-subject variance, individual random effects are less distinguishable from noise. If recovery succeeds under these conservative conditions, it should perform at least as well with the actual data. Binary responses were generated from the logistic function using the manuscript’s fixed-effect estimates. We then fit the identical GLMM to each synthetic dataset and correlated the recovered BLUPs (Best Linear Unbiased Predictors) with the true generating values.

***Results***

All 100 model fits converged successfully. The GLMM recovered participant-level random effects with high fidelity: the median Pearson r between true and recovered random intercepts was .952 (95% simulation interval: [.928, .970]), and for random duration slopes, median r = .932 ([.898, .958]). Fixed-effect estimates were similarly well recovered, with all mean biases < 0.01 and standard deviations closely matching the expected standard errors (see Table S1 and Figure S1). These results demonstrate that 384 trials per participant are more than sufficient for the GLMM to reliably recover both population-level parameters and individual-level random effects.

| **Parameter** | **True** | **Mean Est.** | **SD Est.** | **Bias** |
| --- | --- | --- | --- | --- |
| Intercept | 0.000 | −0.009 | 0.077 | −0.009 |
| Duration | 12.259 | 12.273 | 0.467 | +0.014 |
| Cued brightness | −0.085 | −0.089 | 0.017 | −0.004 |
| Uncued brightness | +0.048 | +0.048 | 0.019 | <0.001 |
| Cued contrast | +0.099 | +0.101 | 0.020 | +0.002 |
| Uncued contrast | −0.006 | −0.005 | 0.017 | +0.001 |

*Table S1.* Fixed-effect recovery across 100 simulations. Bias = Mean Est. − True.


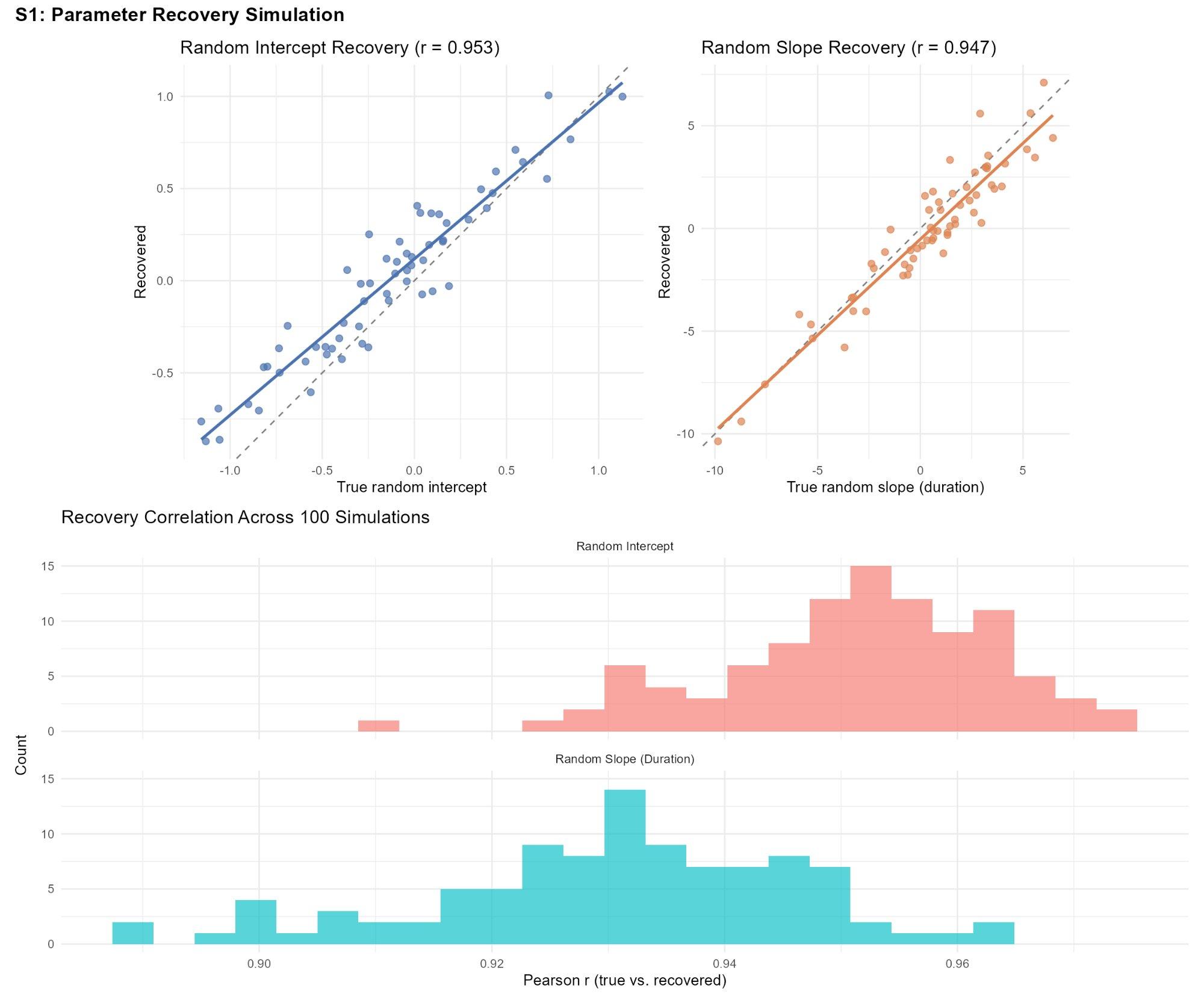


Figure S1**.** Parameter recovery simulation results. *Top row:* Scatter plots of true vs. recovered random intercepts (left; *r* = .953) and random duration slopes (right; *r* = .947) from a representative iteration (closest to median *r*). Dashed line = identity. *Bottom:* Distribution of Pearson *r* between true and recovered BLUPs across 100 Monte Carlo iterations for random intercepts (pink) and random slopes (teal).

## S2. Participant-Level Model Fit Verification

In temporal bisection, scalar timing theory (Gibbon, 1977) predicts that the proportion of ‘long’ responses follows a sigmoid function of probe duration. This arises from the comparison process between the probe and the memorized reference durations with scalar (proportional) noise. The logistic link function in our GLMM is therefore not an arbitrary distributional assumption but the theoretically motivated and empirically established functional form for this task.

***Methods***

To empirically verify that the logistic model captures individual response patterns, we plot observed proportions of ‘long’ responses alongside GLMM-predicted psychometric curves for each of the 63 participants. To maximize the number of observations per data point, we collapsed across brightness and contrast conditions, yielding approximately 60 trials per duration per participant (384 trials ÷ 6 durations ≈ 64 per point, minus excluded trials). We used a simplified model (random intercept + random duration slope, no experimental predictors) so that each participant’s curve reflects only their individual temporal sensitivity and bias.

***Results***

Figure S2 shows the results for all 63 participants. Visual inspection confirms that every participant shows the expected monotonically increasing, sigmoid-shaped psychometric function. No participant exhibits a flat, inverted, or otherwise aberrant response pattern. The GLMM-predicted curves (orange lines) closely track the observed data (blue points) across the full range of probe durations, confirming that the logistic link function provides an adequate description of individual response patterns.

We note that formal per-participant deviance tests are not standard practice within the GLMM framework because individual curves are partially pooled toward the group mean rather than independently fit. Visual verification, combined with the strong theoretical expectation of sigmoidal response curves in temporal bisection, is the accepted approach in the mixed-modeling psychophysics literature (Moscatelli, Mezzetti, & Lacquaniti, 2012).


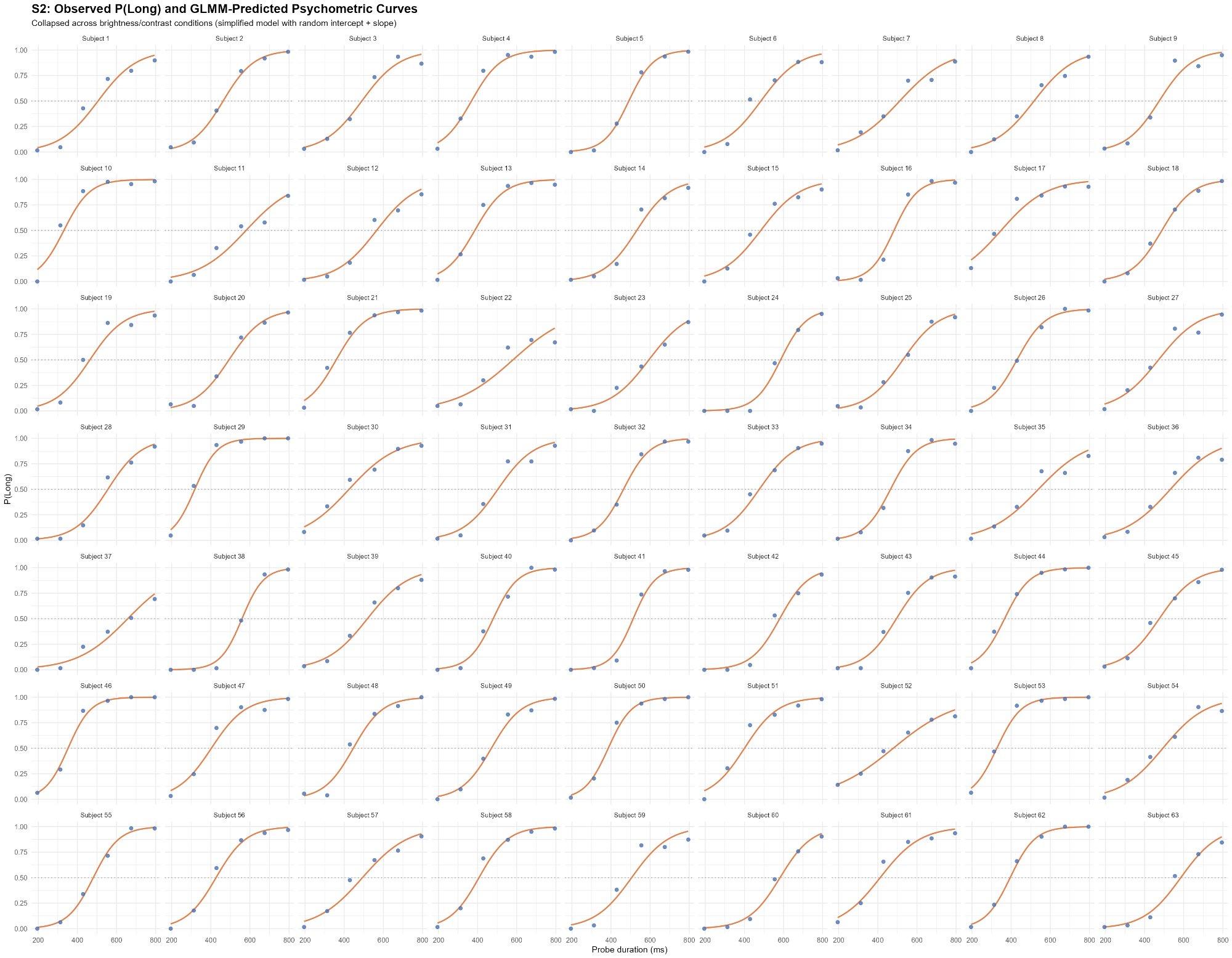


**Figure S2.** Observed P(Long) (blue points) and GLMM-predicted psychometric curves (orange lines) for each of the 63 participants. Data are collapsed across brightness and contrast conditions. The horizontal dotted line marks *P*(Long) = 0.50 (PSE location). All participants show well-behaved sigmoid response functions consistent with scalar timing theory.

## S3. Leave-One-Subject-Out Sensitivity Analysis

***LOSO Methods***

To assess whether any individual participant unduly influences the group-level conclusions, we performed a leave-one-subject-out (LOSO) sensitivity analysis on the *full manuscript model* (including all brightness and contrast predictors and the random intercept + duration slope structure). We refit the GLMM 63 times, each time excluding one participant, and recorded all six fixed-effect coefficients (β_intercept_, β_duration_, β_target brightness_, β_distractor brightness_, β_target contrast_, β_distractor contrast_). All 63 LOSO fits converged successfully.

***LOSO Results***

The duration coefficient (the primary parameter governing the steepness of the psychometric function) was remarkably stable: the full-model estimate was β = 12.259, and across 63 LOSO iterations, values ranged from 12.109 to 12.357 (Max |Δ| = 0.150, or 1.2% of the full-model estimate; see Figure S3). Variance components: τ_int_ = 1.013, τ_slope_ = 3.195, ρ = .399, ICC = .238. The four experimental effects showed comparable absolute stability, with maximum deviations of 0.005–0.008 on the log-odds scale (see Table S2 & Figure S3). The qualitative conclusions—including direction and statistical significance—were unchanged regardless of which participant was excluded. The intercept showed slightly more variation in relative terms (Max |Δ| = 0.050, ~20% of its small absolute value of 0.254), which is expected given that the intercept is the parameter most sensitive to individual response biases. However, this variation in intercept has no bearing on the experimental conclusions, which rest entirely on the brightness and contrast effects and the duration slope.

These results confirm that no single participant drives the group-level findings and that the GLMM’s partial pooling mechanism effectively handles the individual variability present in the data.

| **Coefficient** | **Full model** | **LOSO range** | **Max \|Δ\|** | **Max \|Δ\|%** |
| --- | --- | --- | --- | --- |
| Intercept | 0.254 | [0.204, 0.286] | 0.050 | 19.7%† |
| Duration | 12.259 | [12.109, 12.357] | 0.150 | 1.2% |
| Cued brightness | −0.085 | [−0.092, −0.078] | 0.007 | 8.2% |
| Uncued brightness | +0.048 | [+0.043, +0.054] | 0.005 | 11.2% |
| Cued contrast | +0.099 | [+0.091, +0.106] | 0.008 | 8.1% |
| Uncued contrast | −0.006 | [−0.012, +0.001] | 0.007 | — |

*Table S2.* LOSO stability of fixed-effect estimates. Max |Δ|% is relative to the absolute value of the full-model estimate. The “—” for uncued contrast reflects a near-zero denominator (β = −0.006).


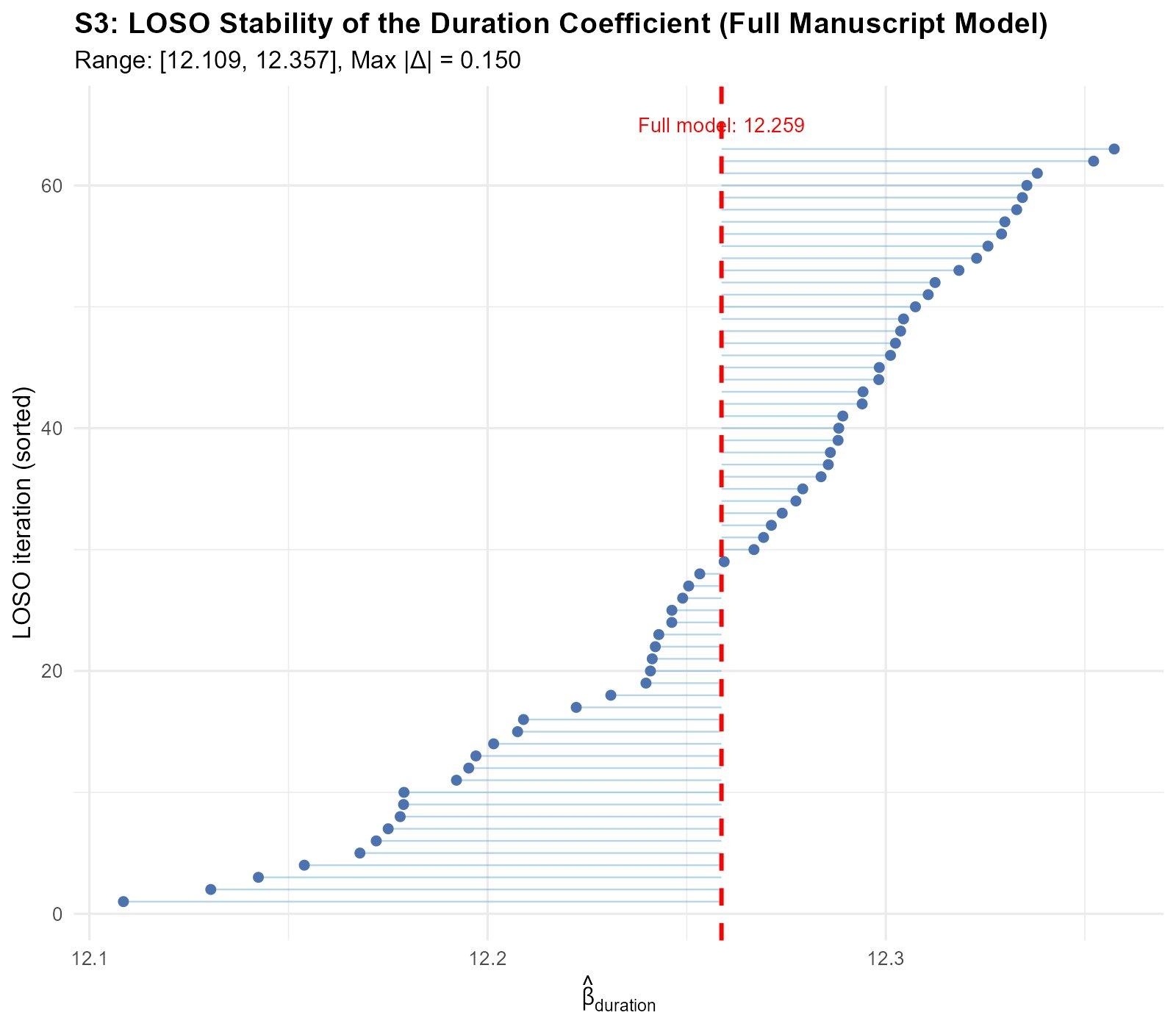


**Figure S3.** Leave-one-subject-out stability of the duration coefficient (β_duration_) from the full manuscript model. Each point represents the estimated coefficient when one participant is excluded. Points are sorted by value; horizontal segments connect each estimate to the full-model value (red dashed line; β = 12.259). The coefficient ranges from 12.109 to 12.357 across 63 iterations (Max |Δ| = 0.150, or 1.2%).

## References

Allan, L. G., & Gibbon, J. (1991). Human bisection at the geometric mean. Learning and Motivation, 22(1–2), 39–58.

Gibbon, J. (1977). Scalar expectancy theory and Weber’s law in animal timing. Psychological Review, 84(3), 279–325.

Moscatelli, A., Mezzetti, M., & Lacquaniti, F. (2012). Modeling psychophysical data at the population-level: The generalized linear mixed model. Journal of Vision, 12(11), 26.

Wearden, J. H., & Ferrara, A. (1995). Stimulus spacing effects in temporal bisection by humans. The Quarterly Journal of Experimental Psychology B, 48B(4), 289–310.
